# Microvascular invasion may be the determining factor in selecting TACE as the initial treatment in patients with hepatocellular carcinoma

**DOI:** 10.1097/MD.0000000000026584

**Published:** 2021-07-09

**Authors:** Joonho Jeong, Jung Gu Park, Kwang Ill Seo, Ji Hyun Ahn, Jae Chun Park, Byung Cheol Yun, Sang Uk Lee, Jin Wook Lee, Jong Hyouk Yun

**Affiliations:** a Division of Gastroenterology and Hepatology, Department of Internal Medicine, Ulsan University Hospital, Ulsan University College of Medicine, Ulsan; b Department of Radiology; c Division of Hepatology, Department of Internal Medicine; d Department of Pathology, Kosin University College of Medicine, Busan, Korea.

**Keywords:** HCC, microvascular invasion, recurrence-free survival, TACE

## Abstract

The aim of this study was to investigate factors affecting tumor necrosis with transcatheter arterial chemoembolization (TACE). Factors associated with early hepatocellular carcinoma recurrence after curative hepatectomy were also evaluated.

Data of 51 patients who underwent surgery after a single session of TACE at a single university hospital were retrospectively analyzed. Factors that might affect tumor necrosis were determined by evaluating the TACE approach and by analyzing computed tomography and TACE findings, pathologic reports, and laboratory findings.

In univariate analysis, microvascular invasion (MVI), radiological capsule appearance on the computed tomography, chronic hepatitis B, diabetes mellitus and serum albumin, MVI were significantly associated with tumor necrosis by TACE (*P* < .02). In multivariate analysis, MVI was the only statistically significant factor in TACE-induced tumor necrosis (*P* = .001). In univariate and multivariate analysis, MVI was the strongest factor for recurrence-free survival rate within 2 years (*P* = .008, *P* = .002).

MVI could be a crucial factor in determining TACE as an initial treatment for hepatocellular carcinoma. MVI is also a strong indicator of recurrence within 2 years after curative hepatic resection.

## 1 Introduction

Curative treatments for hepatocellular carcinoma (HCC) include surgical resection, liver transplantation (LT), and radiofrequency ablation.^[1,2]^ Unfortunately, approximately 75% of HCC patients have metastatic disease or local invasion at the time of diagnosis, making it impossible to perform surgery for these cases. In addition, more than half of patients with an R0 resection could develop recurrence.^[3]^ Overall, HCC is refractory to most treatments in the long term. The 5-year survival rate of patients with HCC has been reported to be as low as 15%.^[1,3]^

Transcatheter arterial chemoembolization (TACE) has mainly been used as a bridge therapy prior to resection or liver transplantation, or in cases of a large and unresectable HCC that is unsuitable for surgery or ablation.^[4]^ A multi-national, multi-center study has announced that TACE is one of the most widely used treatments for HCC in the world regardless of tumor stage. It is also the most frequently selected treatment modality after tumor recurrence.^[5]^ Although the survival advantage offered by TACE is still debatable, 2 randomized controlled trials (RCTs) performed in 2002 have shown that TACE has a survival benefit for patients with unresectable HCC tumors compared to supportive care.^[6,7]^ Several studies have shown that patients with good lipiodol compaction or complete pathologic necrosis after TACE have better overall survival and disease-free survival rates than those who do not obtain necrosis after TACE.^[8–10]^ Other studies have suggested that the presence of both a definite enhancing lesion and a feeding vessel that is >0.9 mm in diameter on a pre-TACE visceral angiogram are associated with an achievement of >90% necrosis on pathology.^[11]^ Furthermore, TACE has been shown to be more effective for 3 to 5-cm tumors than for smaller ones.^[12]^ Another issue with TACE is the technique itself. One group has found that selective TACE is better for tumor necrosis than lobar TACE.^[12]^ Another group has shown that super-selective TACE induces not only complete tumor necrosis, but also peri-tumoral parenchymal necrosis.^[13]^ However, information about factors predicting TACE-induced tumor necrosis for selecting TACE as the initial treatment for patients with HCC is lacking. Thus, the objective of this study was to determine factors affecting conventional TACE-induced tumor necrosis in a group of solitary HCC patients with relatively homogeneous characteristics. Factors that might influence early recurrence of HCC after curative surgical resection were also evaluated.

## 2 Materials and methods

### 2.1 Study design and patients

This study was approved by the Institutional Review Board of Kosin University Gospel Hospital (KUGH) (IRB No. 2020-01-019). The requirement for informed consent from patients was waived. A total of 1135 patients diagnosed with HCC at KUGH between January 1, 2008 and December 31, 2017 were reviewed using their electronic medical records. Seventy-one treatment-naïve HCC patients at KUGH who were newly diagnosed were planned to undergo curative resection after preoperative conventional TACE (cTACE). Of these, 51 patients who underwent preoperative cTACE procedure only once were included. Inclusion criteria were:

(1) age ≥ 18 years;
(2) treatment-naive HCC patients who underwent preoperative TACE only once before resection;
(3) patients who took follow-up liver dynamic computed tomography (CT) at 4 weeks after cTACE; and
(4) patients who had an appropriate postoperative pathology report with information on microvascular invasion (MVI), tumor differentiation, and tumor necrosis extent.

**Figure 1:**
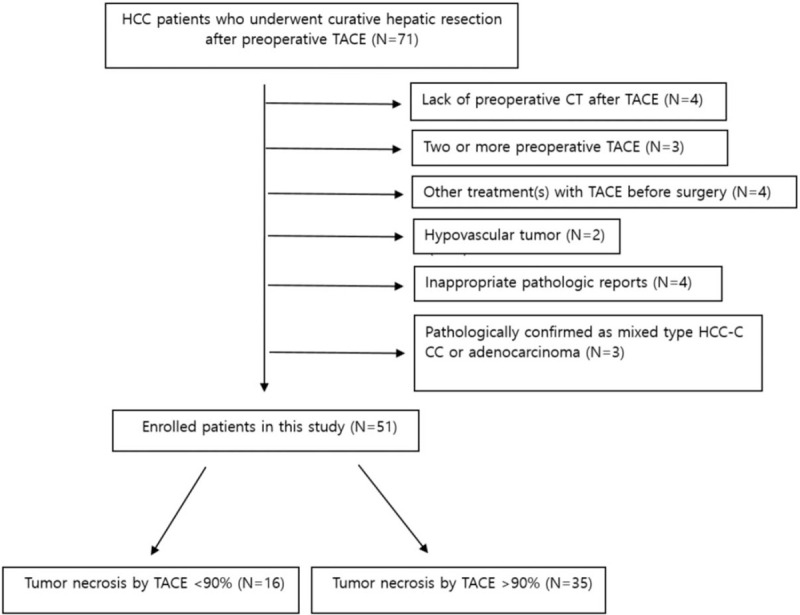
Flow chart showing the initial selection process of the study population.

Twenty patients were excluded from the study. Exclusion criteria were:

(1) those who had preoperative cTACE more than twice;
(2) those who had a cTACE with another loco-regional therapy such as radiofrequency ablation and RT;
(3) patients who had no liver dynamic CT before or after TACE;
(4) patients who had a hypovascular tumor at the time of diagnosis;
(5) patients who was identified as combined HCC-CCC (cholangiocarcinoma) or CCC in the pathology report after hepatic resection;
(6) patients who had pathologic reports without information regarding microvascular invasion or tumor necrosis extent (Fig. 1).

Tumor necrosis extent and MVI were evaluated based on surgical and pathologic reports. We evaluated factors that could affect the extent of tumor necrosis by cTACE by dividing tumor necrosis into groups of 90% or less according to previously published methods.^[12]^ Univariate and multivariate analysis were conducted to identify significant factors. In addition, patients were divided into 2 groups according to TACE approach (super-selective vs non-super-selective approach) and the presence of MVI. Tumor necrosis extents were then compared between different groups of patients.

### 2.2 CT image acquisition

Quadriphasic CT images were acquired using Siemens dual energy CT scanner (Somatom Definition Flash; Siemens Healthcare, Erlangen, Germany). The CT scanning parameters were as follows: detector collimation 128 mm × 0.6 mm, reconstruction at slice thickness of 3 mm and 3-mm slice intervals, and 120 kVp, quality reference 210 mAs for dose modulation system (CareDose 4D; Siemens Medical Solutions). A total of 100 to 150 mL of nonionic contrast medium (350 mgI/mL) was injected at the rate of 4 mL/second through an 18-gauge IV cannula using a power injector. Scan delay was according to an automatic bolus triggering software program (Syngo Acquisition Workplace; Siemens Healthcare, Erlargen, Germany). The late arterial phase scanning and portal venous phase scanning were started at 15 and 55 seconds, respectively, after the trigger threshold was reached (100 Hounsfield units on the abdominal aorta). The delayed phase scanning was performed 180 seconds after the initiation of the contrast material injection.

### 2.3 TACE techniques and imaging diagnosis

Two interventional radiologists with more than 20 years of experience performed cTACE. This study was retrospective in nature. Therefore, we did not dictate the selection of the cTACE procedure. One interventional radiologist performed super-selective TACE while the other one performed non-super-selective TACE depending on their preferences. With the selective approach, tumor-feeding arterial branches were catheterized with a 2.0 Fr-microcatheter. To do so, a catheter was placed into the femoral artery and fed into the hepatic artery. Next, a microcatheter was passed through the previously placed catheter to catheterize tumor-feeding arteries. The tip of the microcatheter was placed within sub-segmental branches of a tumor-feeding artery. This procedure was considered as a super-selective approach. If the tip of the microcatheter was instead placed in a segmental branch, it was considered a selective approach. After the microcatheter was placed, a mixture of iodized oil (Lipiodol; Andre Guerbet, Aulnary-sous-Bois, France) and doxorubicin hydrochloride (Adriamycin; Kyowa Hakko Kogyo, Tokyo, Japan) was infused under fluoroscopic control. Amounts of doxorubicin hydrochloride and lipiodol were determined by the operating interventional radiologist according to the size of the tumor. During a non-selective lobar TACE, a catheter was placed into lobar branches where chemoembolization was performed using the same selective method.

Two other diagnostic radiologists reviewed TACE images, including liver dynamic CT and hepatic angiograms at the time of diagnosis and follow-up CT images after TACE. We analyzed the following radiographic features of MVI as described previously^[14–16]^: non-smooth margin, peri-tumoral arterial enhancement, radiological capsule appearance, and portal venous wash-out.

### 2.4 Pathologic reports of surgical specimens

The extent of tumor necrosis induced by cTACE was evaluated based on pathologic reports of surgical specimens and recorded as a percentage (%). We also evaluated histological differentiation, MVI, and background liver status using pathologic reports. For patients with MVI, another pathologist with 6 years of experience reviewed specimens to validate internal pathological reports. This additional pathologist did not initially evaluate these surgical specimens.

### 2.5 Demographic data and laboratory measurements

Demographic data included the following parameters: age, sex, hepatitis B or C infection, diabetes mellitus, hypertension, and habitual alcohol intake. Patients with more than 60 g of alcohol ingestion per day and more than 3 times per week were considered habitual alcoholics. We also evaluated the following laboratory results: prothrombin time (international normalized ratio), serum albumin, aspartate transaminase, alanine aminotransferase, total/direct bilirubin, alkaline phosphatase, gamma glutamyl transferase, complete blood count, and blood urea nitrogen/creatinine.

### 2.6 Surgical resection

Anatomical resection was performed in most cases. However, wedge resections were also included in the study. Given this study's retrospective nature, we were not involved in the decision regarding the range of resection.

### 2.7 Pre-/postoperative surveillance and early recurrence of HCC after curative resection

A follow-up liver dynamic CT was performed 4 weeks after cTACE. The lipiodol uptake after cTACE was also evaluated on CT. The first follow-up liver dynamic CT was performed within 6 weeks of the surgical resection. After that, the tumor markers and liver dynamic CT were evaluated every 3 months. Positron emission tomography–computed tomography (PET–CT) was performed once a year for the subsequent 2 years. Early recurrence of HCC was defined by that occurring within 2 years of the curative resection.

### 2.8 Statistical analysis

Data was presented as mean values ± standard derivations or percentages. The chi-square test was performed for categorical variables, and the Student *t* test for continuous variables. Univariate and multivariate logistic regression analyses were performed in order to identify factors that may affect the tumor necrosis induced by cTACE and MVI prediction (Tables 2 and 3). Clinical parameters, tumor size,^[17,18]^ CT findings, and serum α-fetoprotein (AFP)^[19,20]^ which were published for predicting MVI were included in the analysis. When comparing the tumor extent between groups, a normality test was performed for each group. Alternatively, the Mann–Whitney test was performed for groups that did not have normality (Supplementary Table 1, Table 2, and Table 3) Recurrence-free survival rate was compared according to the presence of MVI. The Cox regression analysis (univariate and multivariate analysis) was performed including clinical parameters, tumor characteristics, and treatment modality. Univariate and multivariate analyses for factors predictive of TACE-induced tumor necrosis, MVI, and recurrence-free survival were performed using a Cox proportional-hazard model (Table 4). The results of the model were presented as a hazard ratio with a 95% confidence interval. Variables were not included in the multivariate model if *P*-values in the univariate analysis were >0.2. The inter-observer agreement for image analyses of non-smooth margin, peri-tumoral arterial enhancement, radiologic capsule appearance, and portal venous wash-out was assessed by Cohen's kappa. Kappa value was classified as poor (<0.2), fair-to-moderate (ICC, 0.21–0.60), good (0.61–0.80), or very-good (0.81–1.0). These statistical analyses and graph drawing were conducted using SPSS version 25 (SPSS Inc., Chicago, IL) and graph-prism Ver.8-3.

## 3 Results

### 3.1 Baseline clinical and tumor characteristics

The baseline clinical and tumor characteristics are described in Table 1. The average age of the subjects was 61.82 ± 8.33 years. The proportions of men and women were 80.4% and 19.6%, respectively. All patients were of Child-Pugh class A. The rate of hepatitis B surface antigen positivity and anti-hepatitis C virus positivity were 72.5% and 15.7%, respectively. The rate of chronic alcoholism was 33.3%. All patients had a single tumor, with an average diameter of 2.74 ± 1.35 cm. The average serum APF was 326.79 ± 1009.54 ng/mL (Table 1).

**Table 1:** Baseline clinical and tumor characteristics of the study population.

Variables	Values
Age (yrs)	61.82 ± 8.33
Sex (male:female)	41:10 (80.4%:19.6%)
Child-Pugh score A	51 (100%)
ALBI grade I/II	42:9 (82.4%:17.6%)
HBs Ag positivity	37 (72.5%)
Anti-HCV positivity	8 (15.7%)
Alcoholics	17 (33.3%)
Diabetes mellitus	9 (17.6%)
Hypertension	11 (21.6%)
Prothrombin time (INR)	1.06 ± 0.08
Serum albumin (g/dL)	4.15 ± 0.65
Serum total bilirubin (mg/dL)	1.01 ± 0.44
BUN (mg/dL)	14.39 ± 4.44
Serum creatinine (mg/dL)	0.84 ± 0.18
Diameter of tumor (cm)	2.74 ± 1.35
Diameter of tumor below 5 cm	49 (96%)
Diameter of tumor below 3 cm	34 (66.7%)
Serum alpha-fetoprotein (ng/mL)	326.79 ± 1009.54
Super-selective TACE	16 (31.4%)
Selective/lobar TACE	35 (68.6%)
Edmonson grade: III/IV	12:11 (23.5%:21.6%)
Edmonson grade: unknown	26 (51%)

ALBI grade = albumin–bilirubin grade, anti-HCV = antibody to hepatitis C virus, BUN = blood urea nitrogen, HBs Ag = the surface antigen of the hepatitis B virus, INR = international normalized ratio, MELD score = model for end-stage liver disease score, TACE = transarterial chemoembolization.

### 3.2 Comparing tumor necrosis according to TACE approach

In the current study, we first evaluated the difference in TACE-induced tumor necrosis according to TACE approach. Results are shown in Supplementary Table 1 and Table 2. A previously published study has compared selective TACE and non-selective TACE.^[12]^ However, the present study compared tumor necrosis extent between super-selective TACE (sub-segmental TACE) and non-super-selective TACE (lobar/segmental TACE). There were 16 (31.4%) and 35 (68.6%) patients in the super-selective group and the non-super-selective group, respectively. Clinical and tumor characteristics including age, sex, serum AFP, radiological findings, and clinical parameters of each group are shown in Supplementary Table 1. None of these was statistically different variables between the 2 groups. Tumor necrosis extent in each group according to the TACE approach is shown in Supplementary Table 2. Tumor diameters were not significantly different between the super-selective group and the non-super-selective group (2.64 ± 1.34 cm vs 2.69 ± 1.07 cm, *P* = .858). The extent of tumor necrosis induced by TACE was not significantly different between the super-selective and the non-super-selective group either (66.88 ± 43.74% vs 76.23 ± 38.66%, *P* = .148). Rates of complete necrosis and >90% tumor necrosis were also similar between the 2 groups (*P* = .417 and *P* = .569, respectively) (Supplementary Table 2).

### 3.3 Determining factors that could affect tumor necrosis induced by TACE

**Figure 2:**
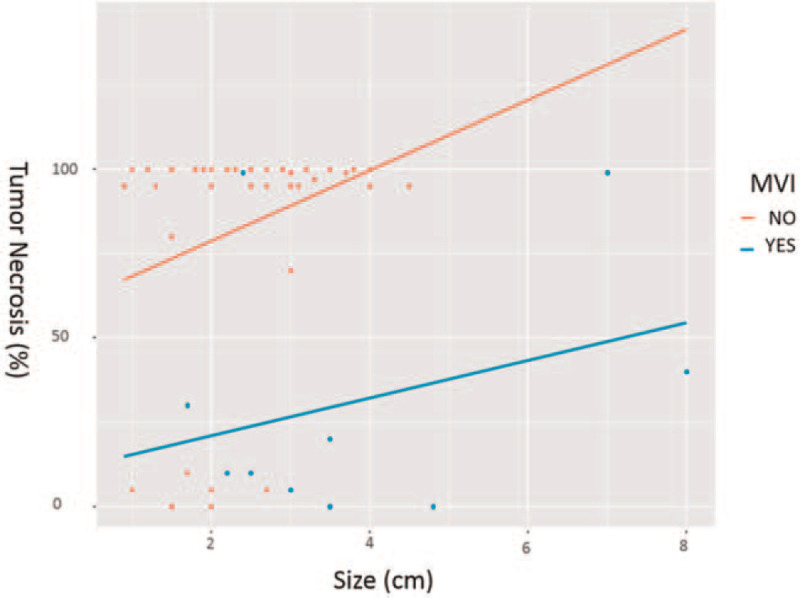
Comparison of tumor necrosis in patients with and without MVI. Changes of tumor necrosis according to the presence of MVI and tumor size. The solid line with 2 different colors is the pendulum line. MVI = microvascular invasion.

The current analysis showed that there was no difference in tumor necrosis according to the TACE approach. Therefore, we performed univariate and multivariate logistic regression analyses to identify factors that might affect tumor necrosis by TACE (Table 2). The following parameters were significantly (*P* < .2) associated with tumor necrosis by TACE in the univariate analysis: MVI, radiological capsule appearance on CT, chronic hepatitis B, DM, and serum albumin. However, in multivariate analysis, MVI was the only statistically significant factor associated with TACE-induced tumor necrosis. These MVIs were identified on postoperative pathologic reports as described in the Section 2. Additionally, when tumor necrosis extent was compared between the groups according to the presence of MVI (Supplementary Table 3), the extent of tumor necrosis by TACE, proportion of patients with complete necrosis, and proportion of patients with >90% of tumor necrosis were lower in the MVI group than those in the non-MVI group (*P* = .001, *P* = .01, and *P* = .001, respectively). Liver dynamic CT performed after TACE also revealed that lipiodol uptake was lower in the MVI group than that in the non-MVI group (*P* = .007). Figure 2 graphically shows changes in tumor necrosis according to the presence of MVI and the size of the tumor.

**Table 2:** Univariate and multivariate logistic regression of factors affecting incomplete tumor necrosis.

	Univariate analysis		Multivariable analysis	
CT findings and tumor characteristics	Hazard ratio (95% CI)	*P*	Hazard ratio (95% CI)	*P*
TACE approach	1.50 (0.430–5.235)	.525		
MVI	16.50 (2.921–93.195)	.002	19.818 (3.322–118.234)	.001
Satellite nodule	2.583 (0.555–12.023)	.226		
Non-smooth margin	0.727 (0.191–2.771)	.641		
Peri-tumoral enhancement	0.000	.999		
Radiological capsule	4.848 (0.992–23.701)	.051	1.549 (0.176–13.615)	.693
Wash-out at portal phase	0.494 (0.137–1.782)	.281		
Age	0.990 (0.921–1.063)	.775		
Tumor size	0.962 (0.621–1.489)	.861		
Serum AFP > 100	1.316 (0.396–4.380)	.654		
Chronic hepatitis B	0.321 (0.089–1.167)	.084	0.377 (0.068–2.101)	.266
Chronic hepatitis C	2.583 (0.555–12.023)	.226		
Diabetes Mellitus	0.225 (0.026–1.976)	.178	0.255 (0.023–2.803)	.264
Hypertension	1.333 (0.328–5.419)	.688		
Chronic alcoholics	1.309 (0.379–4.517)	.670		
PT (INR)	81.804 (0.027–48719.15)	.282		
Total bilirubin	1.467 (0.351–6.128)	.599		
Serum albumin	2.340 (0.680–8.048)	.177	2.926 (0.680–12.587)	.149
ALBI grade (A or B)	0.897 (0.194–4.151)	.889		

AFP = α-fetoprotein, ALBI grade = albumin–bilirubin grade, MVI = microvascular invasion, PT (INR) = prothrombin time (international normalized ratio), TACE = transarterial chemoembolization.

### 3.4 CT findings and clinical parameters predicting MVI in HCC

**Figure 3:**
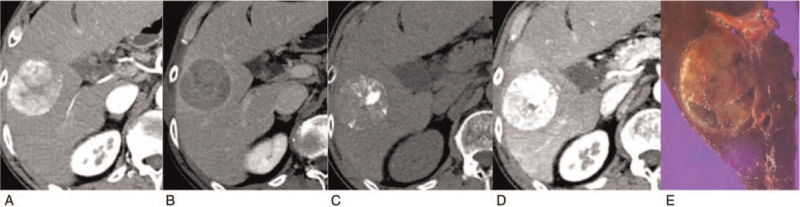
An 80-year-old male with microvascular invasion. (A) Late-hepatic arterial phase image showing heterogeneously hyper-enhancing mass in segment V; (B) on 3-min delayed phase image, the mass shows low attenuation compared to background parenchyma and has capsule appearance; (C and D) on pre- and late-hepatic arterial phase of contrast-enhanced CT at 20 d after cTACE, there is a partial lipiodol accumulation in HCC; (E) gross pathology photograph of resected specimen confirming HCC with microvascular invasion, but no necrosis. HCC = hepatocellular carcinoma, TACE = transarterial chemoembolization.

Table 3 shows predictability for MVI at the time of diagnosis for HCC using CT findings and clinical parameters. Kappa values for CT readings are also provided. Radiological capsule appearance, wash-out at portal phase, tumor size, and serum AFP > 100 ng/mL that had *P* < .2 in univariate analysis were included in the multivariate analysis. Radiological capsule appearance and wash-out at portal phase failed to show statistical significance in the multivariate analysis (*P* = .075 and *P* = .538, respectively). Serum AFP levels could not reliably predict MVI either (*P* = .052). Tumor size was the only parameter that was significantly associated with the presence of MVI (hazard ratio: 2.558, 95% confidence interval: 1.139–5.745, *P* = .023). CT findings and gross pathologic pictures are presented in Figure 3.

**Table 3:** Univariate and multivariate logistic regression of CT findings and clinical parameters predicting MVI of HCC.

	Univariate analysis		Multivariable analysis		
CT findings and tumor characteristics	Hazard ratio (95% CI)	*P*	Hazard ratio (95% CI)	*P*	Kappa value
Non-smooth margin	3.10 (0.121–12.953)	.121			0.636
Radiological capsule	6.167 (1.205–31.550)	.029	5.818 (0.838–40.414)	.075	0.703
Portal venous wash-out	4.179 (0.478 -36.530)	.196	2.118 (0.194–23.096)	.538	0.902
Peri-tumoral arterial enhancement	0	.999			0.847
Tumor size	2.122 (1.125–4.000)	.020	2.558 (1.139–5.745)	.023	
Serum AFP level > 100	2.893 (0.699–11.972)	.143	7.664 (0.980–59.910)	.052	
Age	1.042 (0.955–1.136)	.356			
Serum albumin	0.928 (0.263–3.270)	.907			
PT (INR)	2.551 (0.00–3163.204)	.840			
Total bilirubin	1.963 (0.315–2.230)	.470			
Chronic alcoholics	0.827 (0.185–3.699)	.803			

AFP = α-fetoprotein, CT = computed tomography, HCC = hepatocellular carcinoma, PT(INR) = prothrombin time (international normalized ratio).

### 3.5 MVI can predict the early recurrence of HCC

**Figure 4:**
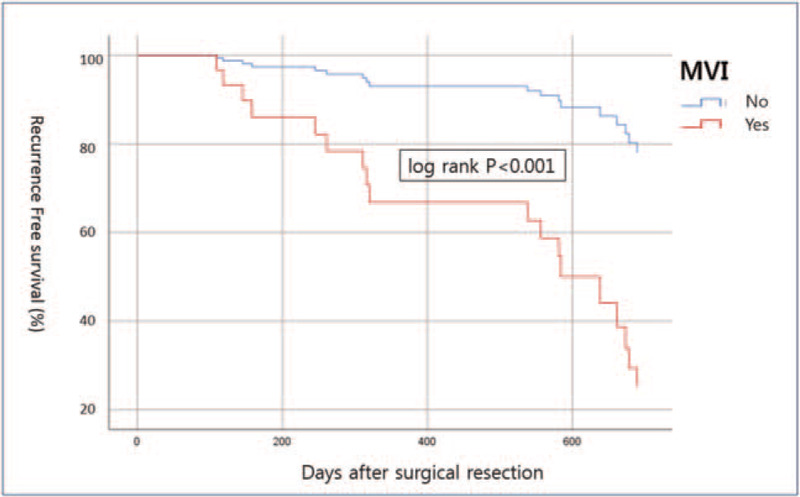
Recurrence-free survival rate based on the presence of MVI. MVI = microvascular invasion.

A prior study has found that patients with early recurrence of HCC have poorer prognosis than those with late recurrence.^[21]^ In this study, early recurrence was defined if the recurrence occurred within 2 years after curative resection. Factors that might affect recurrence-free survival within 2 years of curative resection were analyzed using univariate and multivariate regression analyses. Treatment modalities, clinical parameters, and tumor characteristics were used as variables in these analyses. MVI, satellite nodule, and tumor necrosis extent were confirmed by pathologic reports of surgical resection. In univariate and multivariate analyses, MVI was the strongest factor for recurrence-free survival rate within 2 years (Table 4). Recurrence-free survival rate in this study is presented based upon the presence of MVI (Fig. 4).

**Table 4:** Univariate and multivariate analyses for recurrence-free survival within 2 yrs.

	Univariate analysis		Multivariable analysis	
Variables	Hazard ratio (95% CI)	*P*	Hazard ratio (95% CI)	*P*
TACE approach	0.206–2.722	.660		
MVI (microvascular invasion)	2.668–632.685	.008	2.815–107.000	.002
Tumor necrosis by TACE (%)	1.006–1.081	.022	1.006–1.055	.016
Post-TACE lipiodol uptake (%)	0.962–1.005	.132	0.969–1.000	.057
Anatomical resection	0.1725–4.571	.885		
Satellite nodule	0.074–5.380	.675		
Age	0.891–1.085	.740		
Gender	0.333–11-707	.453		
Tumor size (cm)	0.452–1.485	.511		
Serum AFP level (ng/mL)	0.371–7.468	.505		
The date between TACE and surgical resection (d)	0.990–1.005	.499		
ALBI grade	0.147–3.989	.750		

AFP = α-fetoprotein, ALBI grade = albumin–bilirubin grade, MVI = microvascular invasion, TACE = transarterial chemoembolization.

## 4 Discussion

In current guidelines regarding treatment of HCC, TACE is recommended^[2,22]^ if liver function is preserved while surgical resection and liver transplantation are impossible. However, the effect of TACE on tumor necrosis, the benefit of recurrence-free survival, and the overall survival rate in patients who have undergone TACE remain unclear.^[4]^ In the 2011 Cochrane review, TACE was not recommended as a standard therapy for HCC until more randomized controlled studies showed firmer evidence in its favor.^[23]^ These conflicting results regarding effects of TACE are probably due to different study designs and uneven study populations. Previous studies^[12,24]^ included patients who had different UNOS (United Network for Organ Sharing) stage or beyond Milan Criteria. Additionally, Lobar cTACE was mostly performed in patients with multiple HCCs.^[12]^ Patients with multiple HCCs or portal vein tumor thrombosis were also included in previous analyses.^[12,24]^ In addition, Lobar cTACE was mostly performed in patients with multiple HCCs.^[12]^ Thus, the prior study reported that the tumor necrosis of selective TACE is superior. However, interpretation of such results should be careful. Moreover, there was no subgroup analysis according to tumor characteristics in the previous study.^[12]^ Compared to previous studies of predictability regarding TACE-induced tumor necrosis, the study population of the current study was highly homogeneous. All patients had a single tumor with Child-Pugh class A. They had no distant metastasis or portal vein thrombosis. Furthermore, in contrast with previous studies that evaluated the effect of TACE radiologically, the current study evaluated the effect of cTACE radiologically and pathologically. Unlike previous studies, we identified tumor necrosis of a single session of cTACE and determined factors that could affect tumor necrosis by cTACE. We analyzed 51 patients who underwent a single session of TACE before surgical resection. Most (96%) patients in the study had single tumor with size < 5 cm. A prior study has found that the super-selective TACE approach can lead to more tumor necrosis than a less selective approach.^[13]^ However, in the present study, although the majority of patients had a single tumor of <5 cm in size, there was no statistically significant difference in the effect of tumor necrosis according to the TACE approach radiologically or pathologically. In multivariate analysis, MVI was revealed to be the most crucial factor for TACE-induced tumor necrosis. Patients with MVI were found to have significantly less tumor necrosis than patients without MVI. As shown in Figure 2, there was a clear difference in tumor necrosis according to the presence of MVI. Additionally, there was a noticeable difference in recurrent free survival between the MVI group and the no MVI group, similar to results of preceding studies.

In real clinical situations, TACE is performed repeatedly with or without other treatment modalities when the tumor necrosis is insufficient after the initial TACE procedure. However, repeated TACE procedures not only can lead to adverse side effects such as vascular or bile duct injury and hepatic dysfunction,^[25]^ but also can lead to TACE refractoriness with little or no additional tumor necrosis.^[4,26]^ For these reasons, how long TACE should be used clinically and when it should be replaced with other treatments remain unclear. The problem with TACE refractoriness is that there is no further tumor necrosis. In addition, if incomplete pathologic necrosis is induced, it may lead to more aggressive tumor biology due to hypoxic damage of embolization.^[27]^ Therefore, TACE refractoriness could actually adversely affect a patient's prognosis. A previous study of 314 treatment-naïve patients has shown that complete response after the first TACE is the most robust predictor of a favorable outcome in HCC.^[28]^ A study of 490 patients with unresectable HCCs has demonstrated that patient's radiological response after TACE could predict prognosis. In that study, the initial compact lipiodol uptake is an independent predictor of improved survival rate.^[10]^ Several prior studies have shown that patients who develop complete pathological necrosis after TACE have better overall survival and disease-free survival rates than those who do not develop complete necrosis.^[8,9,29]^ Another study has suggested a model that could predict complete pathological response, showing that in patients with a tumor < 3 cm in size, a preoperative AFP < 100 ng/mL, and a single tumor, the accuracy of complete pathologic response could be predicted approximately 47.9%.^[9]^ Data regarding how to predict the effect of TACE proactively are lacking, although several studies have predicted TACE refractoriness in different ways.^[26,30–32]^ Thus, an indicator is needed to preemptively predict the effect of TACE and recognize TACE refractoriness. For these reasons, the result of the current study showing that MVI can be a determining factor for incomplete tumor necrosis by TACE would be of critical importance clinically. The prediction of MVI prior to TACE would have a significant impact clinically in real practice.

Over the past decade, microvascular invasion has been recognized as a crucial histopathologic prognostic factor of HCC.^[33]^ HCC patients with MVI are known to have a significantly higher rate of recurrence within 2 years after surgical resection or liver transplantation than those without MVI.^[20,34–36]^ In addition, recurrence-free survival and overall survival rates differ according to the range and extent of MVI.^[37–39]^ Therefore, several prior studies have performed MVI prediction at the time of preoperative diagnosis. MVI predictions have been attempted using tumor size and tumor markers^[17,18,40–42]^ as well as findings on CT,^[14–16,19,43]^ gadolinium MRI,^[18,44,45]^ and PET–CT.^[46,47]^ On CT imaging, MVI has been associated with non-smooth margins and radiological capsule disruption. In the case of MRI, MVI can be predicted with high diagnostic accuracy when there are arterial peri-tumoral enhancement, non-smooth tumor margin, and peri-tumoral hypointensity at the hepatobillary phase (when 2 of these 3 findings were combined, 52.4% of HCCs with MVI were identified with a specificity of 92.5%).^[45]^ Another study has reported that MRI findings could predict not only MVI, but also aggressive tumor biology (peri-tumoral hypointensity adjusted odds ratio 2.99, *P* = .002 and satellite nodule adjusted odds ratio 7.972, *P* = .006).^[44]^ On PET–CT, tumor to normal liver standardized uptake value ratio (TLR) and 18F-fluorodeoxyglucose (18F-FDG) uptake might be helpful for predicting MVI (TLR on TDF PET–CT hazard ratio 2.43, *P* = .047 and 18F-FDG uptake hazard ratio 13.4, *P* = .001).^[46,47]^ However, several precautions must be considered when applying such MVI predictions to clinical scenarios. For instance, more external validation is needed for imaging modalities. Inter-observer variability is one main issue. In addition, tumor markers used in other studies might be different and their cut-off values might be inconsistent 40 to 42.^[17,18,40–42]^ Unlike preceding studies that attempted MVI prediction using liver dynamic CT or tumor markers, it was infeasible to predict MVI statistically using CT findings or tumor markers in the present study. It was only possible to confirm results of previous studies that MVI and tumor size were related to each other. This result was contrary to the relatively good MVI predictions with preoperative gadolinium MRI. Nonetheless, the current study revealed that the presence of MVI reported to play an important role in postoperative outcomes in patients with HCC could also be a crucial factor for predicting tumor necrosis effects of TACE.

MVI was found to be the most important factor in early recurrence-free survival after curative resection in the present study, consistent with previous studies.^[37,38]^ Although the role of adjuvant therapy after curative resection has not been established, poor prognosis of early recurrence of HCC and the result of MVI as an excellent predictor of tumor recurrence^[48]^ suggest that adjuvant therapy is necessary for certain patients. In other words, the presence of MVI could inform the necessity of adjuvant therapy after curative resection. Recently, a few studies including some small randomized controlled trials have reported that adjuvant TACE,^[49]^ radiotherapy,^[50]^ and sorafenib^[51]^ can improve the disease-free survival rate of MVI-positive patients. Therefore, the role of adjuvant therapy must be confirmed through large-scale observation studies or randomized controlled trials.

The current study is the first to assess the association of TACE effectiveness with MVI in HCC patients. The strength of this study was that the analysis was based upon a homogenous study population, rendering reliable effect of TACE. Another strength of the current study was that pathological analysis was conducted in conjunction with radiological assessment. Nonetheless, this study also has a few limitations. First, this study was a retrospective observation study. Thus, it might be subjected to selection bias. Second, this study had a small sample size. More subjects are needed to have to a greater statistical power. Nonetheless, we made a homogenous study population and analyze the subjects radiologically and pathologically. Third, this single-center study might be limited to generalization of results.

In conclusion, we found that MVI was an important factor in incomplete tumor necrosis of TACE. Results of the current study suggest that TACE is not an ideal initial treatment for HCC patients with MVI. In recent studies, HCC with MVI can be predicted using several imaging modalities, especially gadolinium MRI; therefore, it may be recommended to determine whether to conduct TACE as the first treatment of HCC depending on the possibility of MVI. Moreover, clinicians need to be cautious when selecting TACE for HCC patients with MVI in intermediate stage or in the setting of bridge therapy or down-staging before LT and curative resection. In the future, well-designed studies are needed to devise a model to predict the efficacy of TACE according to MVI predictions. Prospective studies are also needed to compare outcomes of TACE and other treatment modalities for HCC patients with MVI.

## Author contributions

**Conceptualization:** Joonho Jeong, Jung Gu Park.

**Data curation:** Joonho Jeong, Ji Hyun Ahn, Jae Chun Park.

**Funding acquisition:** Jung Gu Park.

**Investigation:** Jung Gu Park.

**Methodology:** Joonho Jeong, Jung Gu Park.

**Writing – original draft:** Joonho Jeong.

**Writing – review & editing:** Joonho Jeong, Jung Gu Park, Kwang Ill Seo, Byung Cheol Yun, Sang Uk Lee, Jin Wook Lee, Jong Hyouk Yun.

## Supplementary Material

Supplemental Digital Content

## Supplementary Material

Supplemental Digital Content

## Supplementary Material

Supplemental Digital Content
