# Supplementary material for: Microvascular invasion may be the determining factor in selecting TACE as the initial treatment in patients with hepatocellular carcinoma
Source: Medicine (Baltimore). 2021 Jul 9;100(27):e26584. doi: 10.1097/MD.0000000000026584 (PMC8270609; doi:10.1097/MD.0000000000026584)
Supplement: Supplemental Digital Content [file medi-100-e26584-s001.docx]

Supplementary Table1. Clinical and tumor characteristics of subjects according to TACE approach

| **Variables** | **TACE approach** | | **p-value** |
| --- | --- | --- | --- |
|  | **Selective/lobar (N=35)** | **Super-selective  (N=16)** |  |
| Age | 61.86 ± 8.44 | 61.75 ± 8.34 | 0.967 |
| Gender Male | 29 (82.86%) | 12 (75.00%) | 0.783 |
| Female | 6 (17.14%) | 4 (25.00%) |  |
| Size | 2.68 ± 1.49 | 2.86 ± 1.00 | 0.467 |
| AFP (ng/ml) | 391.58 ± 1202.44 | 185.06 ± 297.88 | 0.345 |
| HBsAg Positivity | 25 (71.43%) | 12 (75.00%) | 1.000 |
| Anti-HCV positivity | 5 (14.29%) | 3 (18.75%) | 1.000 |
| Non-smooth margin |  |  | 1.000 |
| no | 25 (71.43%) | 11 (68.75%) |  |
| Yes | 10 (28.57%) | 5 (31.25%) |  |
| Peritumoral enhancement |  |  | 0.783 |
| no | 33 (94.29%) | 14 (87.50%) |  |
| yes | 2 (5.71%) | 2 (12.50%) |  |
| Radiological capsule |  |  | 0.994 |
| no | 29 (82.86%) | 14 (87.50%) |  |
| yes | 6 (17.14%) | 2 (12.50%) |  |
| Portal venous washout |  |  | 0.154 |
| no | 7 (20.00%) | 7 (43.75%) |  |
| yes | 28 (80.00%) | 9 (56.25%) |  |
| DM no | 29 (82.86%) | 13 (81.25%) | 1.000 |
| yes | 6 (17.14%) | 3 (18.75%) |  |
| HTN no | 30 (85.71%) | 10 (62.50%) | 0.133 |
| yes | 5 (14.29%) | 6 (37.50%) |  |
| Alcoholics no | 25 (67.57%) | 12 (66.67%) | 1.000 |
| yes | 12 (32.43%) | 6 (33.33%) |  |
| MELD score | 4.69 ± 3.01 | 5.19 ± 2.95 | 0.581 |
| ALBI grade I | 30 (85.71%) | 12 (75.00%) | 0.592 |
| II | 5 (14.29%) | 4 (25.00%) |  |
| WBC | 5378.57 ± 1198.46 | 5526.88 ± 1329.76 | 0.694 |
| Hemoglobin | 13.88 ± 1.55 | 13.79 ± 1.24 | 0.840 |
| Platelet (K) | 145.11 ± 41.16 | 143.69 ± 59.74 | 0.921 |
| PT (INR) | 1.06 ± 0.08 | 1.08 ± 0.07 | 0.399 |
| Serum total bilirubin | 0.95 ± 0.35 | 1.12 ± 0.60 | 0.297 |
| Serum total protein | 7.39 ± 0.61 | 7.29 ± 0.44 | 0.542 |
| Serum albumin | 4.15 ± 0.74 | 4.12 ± 0.41 | 0.857 |
| BUN | 13.90 ± 4.43 | 15.45 ± 4.40 | 0.251 |
| Serum Cr | 0.85 ± 0.18 | 0.82 ± 0.19 | 0.656 |
| Serum AST | 49.66 ± 52.90 | 80.50 ± 120.20 | 0.338 |
| Serum ALT | 33.34 ± 19.58 | 71.12 ± 97.28 | 0.144 |
| Serum ALP | 68.62 ± 22.96 | 87.69 ± 35.38 | 0.062 |
| Serum r-GTP | 94.69 ± 111.11 | 189.62 ± 253.37 | 0.168 |

*AFP, alpha fetoprotein; DM, diabetes mellitus; HTN, hypertension; WBC, white blood cell; Cr, creatinine; AST, aspartate aminotransferase; ALT, alanine aminotransferase; ALP, alkaline phosphatase; rGTP, gamma glutamyltransferase.*

In the current study, we first evaluated the difference in TACE-induced tumor necrosis according to TACE approach. A previously published study has compared selective TACE and non-selective TACE. However, the present study compared tumor necrosis extent between super-selective TACE (sub-segmental TACE) and non-super-selective TACE (lobar/segmental TACE). There were 16 (31.4%) and 35 (68.6%) patients in the super-selective group and the non-super-selective group, respectively. Clinical and tumor characteristics including age, sex, serum AFP, radiological findings, and clinical parameters of each group are shown in Supplementary Table 1. None of these was statistically different variables between the two groups.
