# Supplementary material for: Microvascular invasion may be the determining factor in selecting TACE as the initial treatment in patients with hepatocellular carcinoma
Source: Medicine (Baltimore). 2021 Jul 9;100(27):e26584. doi: 10.1097/MD.0000000000026584 (PMC8270609; doi:10.1097/MD.0000000000026584)
Supplement: Supplemental Digital Content [file medi-100-e26584-s002.docx]

Supplementary Table 2. Characteristics of nodules of surgical specimens according TACE approach

| **Variables** | **All patients**  **(51 patients)** | **TACE approach** | | **P value** |
| --- | --- | --- | --- | --- |
|  |  | **Selective/Lobar**  **(35 patients)** | **Super-selective**  **(16 patients)** |  |
| Diameters of tumor (Cm) 2.74 ± 1.35 | | 2.69 ± 1.07 | 2.64 ± 1.34 | 0.858 |
| Extent of tumor necrosis (%) 73.29 ± 40.12 | | 76.23 ± 38.66 | 66.88 ± 43.74 | 0.148 |
| Degree of necrosis | |  |  |  |
| Complete necrosis 21 (38.18%) | | 16 (43.24%) | 5 (27.78%) | 0.417 |
| Necrosis > 90% 35 (63.64%) | | 25 (67.57%) | 10 (55.56%) | 0.569 |

Tumor necrosis extent in each group according to the TACE approach is shown in Supplementary Table 2. Tumor diameters were not significantly different between the super-selective group and the non-super-selective group (2.64 ± 1.34 cm vs. 2.69 ± 1.07 cm, *p* = 0.858). The extent of tumor necrosis induced by TACE was not significantly different between the super-selective and the non-super-selective group either (66.88 ± 43.74% vs. 76.23 ± 38.66%, *p* = 0.148). Rates of complete necrosis and > 90% tumor necrosis were also similar between the two groups (*p* = 0.417 and *p* = 0.569, respectively)
