# Supplementary material for: Microvascular invasion may be the determining factor in selecting TACE as the initial treatment in patients with hepatocellular carcinoma
Source: Medicine (Baltimore). 2021 Jul 9;100(27):e26584. doi: 10.1097/MD.0000000000026584 (PMC8270609; doi:10.1097/MD.0000000000026584)
Supplement: Supplemental Digital Content [file medi-100-e26584-s003.docx]

Supplementary Table 3. Clinical Characteristics of subjects with or without MVI of HCC

| **Variables** | **Microvascular invasion (MVI)** | | **p-value** |
| --- | --- | --- | --- |
|  | **MVI positive (N=10)** | **MVI-negative  (N=41)** |  |
| Age | 64.00 ± 9.99 | 61.29 ± 7.92 | 0.362 |
| Gender Male | 9 (90.00%) | 32 (78.05%) | 0.682 |
| Female | 1 (10.00%) | 9 (21.95%) |  |
| Serum AFP (ng/ml) | 263.71 ± 251.92 | 342.17 ± 1121.81 | 0.685 |
| HBsAg Positivity | 6 (60.00%) | 31 (75.61%) | 0.551 |
| Anti-HCV positivity | 3 (30.00%) | 5 (12.20%) | 0.366 |
| Diabetes Mellitus |  |  | 0.807 |
| no | 9 (90.00%) | 33 (80.49%) |  |
| Yes | 1 (10.00%) | 8 (19.51%) |  |
| Hypertension |  |  | 0.573 |
| no | 9 (90.00%) | 31 (75.61%) |  |
| yes | 1 (10.00%) | 10 (24.39%) |  |
| Alcoholics |  |  | 1.000 |
| no | 7 (70.00%) | 27 (65.85%) |  |
| yes | 3 (30.00%) | 14 (34.15%) |  |
| PT (INR) | 1.06 ± 0.08 | 1.06 ± 0.08 | 0.402 |
| Serum Albumin (mg/dl) | 4.19 ± 0.45 | 4.13 ± 0.69 | 0.810 |
| MELD score | 4.60 ± 1.90 | 4.90 ± 3.19 | 0.776 |
| ALBI grade I | 9 (90.00%) | 33 (80.49%) | 0.807 |
| II | 1 (10.00%) | 8 (19.51%) |  |

*AFP, alpha fetoprotein; HBsAg, hepatitis B virus surface antigen; PT(INR), prothrombin time (international normalized ratio); MELD score, Model for End-stage Liver Disease score; ALBI grade, Albumin-Bilirubin grade.*

Supplementary Table 3. None of these was statistically different variables between the two groups
